# How Big Data, Comparative Effectiveness Research, and Rapid-Learning Health-Care Systems Can Transform Patient Care in Radiation Oncology

**DOI:** 10.3389/fonc.2018.00155

**Published:** 2018-05-09

**Authors:** Jason C. Sanders, Timothy N. Showalter

**Affiliations:** Department of Radiation Oncology, University of Virginia School of Medicine, Charlottesville, VA, United States

**Keywords:** big data, radiation oncology, comparative effectiveness research, rapid-learning health care system, personalized radiation therapy

## Abstract

Big data and comparative effectiveness research methodologies can be applied within the framework of a rapid-learning health-care system (RLHCS) to accelerate discovery and to help turn the dream of fully personalized medicine into a reality. We synthesize recent advances in genomics with trends in big data to provide a forward-looking perspective on the potential of new advances to usher in an era of personalized radiation therapy, with emphases on the power of RLHCS to accelerate discovery and the future of individualized radiation treatment planning.

## Comparative Effectiveness Research (CER) and Big Data

The Committee on CER Prioritization was created by the Institute of Medicine in 2009. They defined CER as “a strategy that focuses on the practical comparison of two or more health intervention to discern what works best for which patients and populations” (1). In essence, the goal of CER is to help answer the question “which treatment will work best, in which patient, under what circumstances?” (2). Big Data refers to data sets that are so large that they cannot be analyzed directly by individuals or traditional processing software. Big Data Analytics (BDA) is a growing field with a multitude of methods that is being utilized in various sectors from business to medicine (3). The advent of the Electronic Medical Record (EMR) has resulted in the digitalization of massive data sets of medical information including: clinic encounters, laboratory values, imaging data sets and reports, pathology reports, patient outcomes, family history, genomic, and biological data, etc.

To help with the analysis of Big Data, the NIH has created the Big Data to Knowledge (BD2K) program which has invested over $200 million in grant awards to foster the development of methods and tools to analyze Big Data in biomedical research (4). Additionally, the BD2K program will move to make sure that biomedical Big Data is “Findable, Accessible, Interoperable, and Reusable” (4). Over the past decade, CER methodologies have become increasingly prevalent in radiation oncology research and there is much enthusiasm surrounding BDA.

## Rapid-Learning Health Care System (RLHCS) and Personalized Medicine

The number of articles on Big Data in health care has increased exponentially from under 500 articles in 2005 to over 2500 articles in 2015 (5). As the amount of biomedical Big Data and our ability to analyze these data continues to advance, so will the implications and utilizations of the information we are able to extract. One of the most important steps toward advancing our ability to analyze these Big Data for biomedical discovery is the creation of RLHCS, which will allow for the sharing of patient data between EMRs, ideally in real-time (6). An ideal RLHCS would take patient data that was routinely generated as part of standard patient care and compile that data into a large data system (6–8). This aggregate data would then be available for both BDA to accelerate identification of new hypotheses and CER to rapidly generate evidence through hypothesis-testing studies. Clinical data from patient records can be used readily to identify novel relationships among clinical factors and patient outcomes, or to evaluate treatment effectiveness in specific subgroups, that cannot be studied adequately in randomized, controlled trials. The extreme power of RLCHS, though, is even more exciting when one considers the possibility of adding biospecimens to accelerate discovery in genomics and proteomics. As RLHCS are created and their data sets are expanded, we will continue to identify specific genomic and proteomic data to help define cohorts and stratify patients into risk groups, treatment response groups, and potentially to help design highly tailored therapy regimens (9). In this sense, RLCHS would usher in a more fertile era for improving biomedical research than ever before. BDA and CER provide the research methodologies needed to rapidly generate evidence using RLHCS. It should be noted, however, that there are substantial practical obstacles that must be addressed to achieve the vision of RLHSC. These include patient concerns regarding privacy and security of sensitive information, interconnectivity among different health records, and regulatory barriers to the exchange of health information.

## Integrating a RLHCS with Oncology

The integration of CER, Big Data, and BDA is especially important in the field of Oncology where multiple groups are investing significant time and resources in efforts to expand the availability of data and advance the methods used to extract meaningful information from that data (4, 10–14). The American Society of Clinical Oncology started their own RLHCS, CancerLinQ, to overcome the lack of interoperability between EMRs and accomplish their goal of being able to “analyze and share data on every patient with cancer” (15). While the vision of RLCHS has not yet been fully achieved, the potential impact on society has stimulated enthusiasm toward this effort.

## Implications for Radiation Oncology

### Patient Reported Outcomes (PROs)

Patient reported outcomes and quality-of-life (QoL) have become a major area of focus in health care overall, particularly in oncology. The availability of PROs within EMRs provides the foundation for a RLHCS that can be leveraged to expand insights into how cancer treatments impact patient QoL. By incorporating the PROs for massive numbers of patients, RLHCS will be able to identify small variations and subgroups of patients that might be missed in the smaller number of patients included in traditional randomized controlled trials. These PROs and QoL domains can then be incorporated into clinical decision-making to help guide both providers and patients (16). In doing this, PROs can act as a link between the objective clinical data and the subjective patient outcomes and experiences to help improve the overall care of the patient (17). One may also conceive of potential genomics-based determinants of QoL that could be identified using BDA if RLHCS include biospecimens linked to clinical data and PROs. Finally, surveillance of a RLHCS may also be performed to identify temporal trends in PROs to estimate outcomes after implementation of new technologies.

### Dose Selection and Radiosensitivity

The use of tumor-specific genes and radiosensitivity to guided treatment decisions has already been established in human papilloma virus-associated squamous-cell carcinoma of the oropharynx (18). Numerous studies have looked at identifying genes that may have implications on tumor radiosensitivity or patient toxicity (19–22). The identification of these genes and their potential implications has led to the creation of the fields of radiogenetics and radiogenomics. Efforts are currently underway to generate meaningful gene assays that will help predict tumor response to radiation. Eschrich et al. created a 10-gene model to calculate a radiosensitivity index and applied this to patients with head-and-neck, rectal, and esophageal cancer to help stratify patients into either responders or non-responders with 80% sensitivity and 82% specificity (22). Similarly, Zhao et al. retrospectively created a 24-gene assay and applied this to risk matched patients who either received postoperative radiation or no radiation following prostatectomy. Patients with a high score on the gene index who received postoperative radiation were less likely to have distant metastasis at 10 years (23). As efforts to identify genes and gene assays that may be predictors of radiosensitivity continue to be validated, we will potentially be able to integrate these findings in dose selection and toxicity prediction for individual patients based on their native and tumor genetics. Scott and colleagues have recently described a genomics-based strategy for personalizing radiation therapy dose, which would support dose de-escalation for radiosensitive tumors (24). While the clinical implication of radiosensitivity assays are still developing, big data will be key to developing future assays rapidly, as well as incorporating the genomics tools into clinical decision-making. Big data provides opportunity to refine molecular signatures based upon real-world data and to merge genomic assay results with other clinical data elements to optimize predictive analytics. A RLHCS would provide the ideal substrate for levering big data and CER to accelerate genomics-based discovery to make precision radiation oncology a reality.

### Personalized Treatment Recommendations

Radiation oncology is unique in that treatment plans for patients are often already technically and physically personalized due to patient-specific variations in anatomy, tumor characteristics, and stage. Since a patient’s treatment plan is usually based upon a CT scan in treatment position, radiation can be considered an inherently personalized form of medicine. However, treatment planning approaches and radiation doses are generally selected based upon class solution, with technical details such as beam arrangements and dose–volume constraints adherent to generalized rules. Multiple studies have already begun to look at how BDA methods such as machine learning and neural networks can be used to aid in dose optimization and toxicity prediction modeling in radiation oncology (17, 25–27), which could provide more optimal treatment plan alternatives for individual patients. As the data and technology behind RLHCS continues to progress, we will likely be able to utilize a full spectrum of patient-specific clinical factors, PROs, genomics, patient preference, and priorities, and a menu of treatment plan alternatives in order to optimize an individual patient’s radiation therapy. In order to deliver high-quality, high impact insights into radiation oncology, it is important that large datasets include detailed technical.

## Conclusion

Much of the excitement regarding big data has centered on potential for genomic discovery, high-level radiation treatment planning, and leveraging EMRs to identify associations among factors that may provide new insights into potential causal relationships that can be further studied to accelerate progress in cancer care. Although these are certainly promising areas for discovery, we most eagerly anticipate the power of big data to connect a broad range of characteristics to accelerate evidence generation and inform personalized decision-making. We envision the use of big data and CER methods to inform the individual decisions of patients and providers by synthesizing clinical and genomic data and querying a RLHCS for the latest data on effectiveness of treatment options in relevant subgroups of patients.

## Author Contributions

Both authors contributed to the development and editing of the manuscript and approved the final submitted version.

## Conflict of Interest Statement

The authors declare that the research was conducted in the absence of any commercial or financial relationships that could be construed as a potential conflict of interest.
